# One-pot bioethanol production from cellulose by co-culture of *Acremonium cellulolyticus* and *Saccharomyces cerevisiae*

**DOI:** 10.1186/1754-6834-5-64

**Published:** 2012-08-31

**Authors:** Enoch Y Park, Kazuya Naruse, Tatsuya Kato

**Affiliations:** 1 Laboratory of Biotechnology, Integrated Bioscience Section, Graduate School of Science and Technology, Shizuoka University, 836 Ohya, Suruga-ku, Shizuoka, 422-8017, Japan; 2 Laboratory of Biotechnology, Faculty of Agriculture, Department of Applied Biological Chemistry, Shizuoka University, 836 Ohya, Suruga-ku, Shizuoka, 422-8017, Japan

**Keywords:** Bioethanol, Cellulase, Biomass, *Acremonium cellulolyticus* C-1, *Saccharomyces cerevisiae*, Biorefinery

## Abstract

**Background:**

While the ethanol production from biomass by consolidated bioprocess (CBP) is considered to be the most ideal process, simultaneous saccharification and fermentation (SSF) is the most appropriate strategy in practice. In this study, one-pot bioethanol production, including cellulase production, saccharification of cellulose, and ethanol production, was investigated for the conversion of biomass to biofuel by co-culture of two different microorganisms such as a hyper cellulase producer, *Acremonium cellulolyticus* C-1 and an ethanol producer *Saccharomyces cerevisiae*. Furthermore, the operational conditions of the one-pot process were evaluated for maximizing ethanol concentration from cellulose in a single reactor.

**Results:**

Ethanol production from cellulose was carried out in one-pot bioethanol production process. *A. cellulolyticus* C-1 and *S. cerevisiae* were co-cultured in a single reactor. Cellulase producing-medium supplemented with 2.5 g/l of yeast extract was used for productions of both cellulase and ethanol. Cellulase production was achieved by *A. cellulolyticus* C-1 using Solka-Floc (SF) as a cellulase-inducing substrate. Subsequently, ethanol was produced with addition of both 10%(v/v) of *S. cerevisiae* inoculum and SF at the culture time of 60 h. Dissolved oxygen levels were adjusted at higher than 20% during cellulase producing phase and at lower than 10% during ethanol producing phase. Cellulase activity remained 8–12 FPU/ml throughout the one-pot process. When 50–300 g SF/l was used in 500 ml Erlenmeyer flask scale, the ethanol concentration and yield based on initial SF were as 8.7–46.3 g/l and 0.15–0.18 (g ethanol/g SF), respectively. In 3-l fermentor with 50–300 g SF/l, the ethanol concentration and yield were 9.5–35.1 g/l with their yields of 0.12–0.19 (g/g) respectively, demonstrating that the one-pot bioethanol production is a reproducible process in a scale-up bioconversion of cellulose to ethanol.

**Conclusion:**

*A. cellulolyticus* cells produce cellulase using SF. Subsequently, the produced cellulase saccharifies the SF, and then liberated reducing sugars are converted to ethanol by *S. cerevisiae*. These reactions were carried out in the one-pot process with two different microorganisms in a single reactor, which does require neither an addition of extraneous cellulase nor any pretreatment of cellulose. Collectively, the one-pot bioethanol production process with two different microorganisms could be an alternative strategy for a practical bioethanol production using biomass.

## Background

A co-culture as a mimic of natural environment has been used for biodegradation of aromatic compounds [1,2] or for biological reduction of sulfate [3]. In addition, ethanol production from a mixture of glucose and xylose was applied using co-cultures of *Pichia stipites* with *Zymomonas mobilis*[4] or *Saccharomyces cerevisiae*[5]. Thus the co-culture is a potential bioprocess if there are no cross-interactions among microorganisms, and each microorganism metabolizing its substrate is unaffected by the presence of other microorganism.

To prepare lignocellulose for ethanol production, the substrate is normally either hydrolysed completely to the reducing sugars using mineral acids or solubilized with a milder pretreatment, leaving the residual cellulose to be saccharified enzymatically. For a conversion of this cellulose to ethanol, either diluted-acid hydrolysate [6] or cellulase-saccharified hydrolysate [7] was used for the co-culture using yeast with *Escherichia coli* or *Z. mobilis*, respectively. When acid-hydrolysate is used, detoxification of the inhibitory components is required to increase the yield. In addition, when glucose and xylose are converted by co-culture using different microorganisms to ethanol production, the saccharification process is still essentially required. Alternatively a one-step process, a combination of cellulase production, cellulose hydrolysis, and fermentation, was applied in the co-culture of *Clostridium thermocellum* and *Z. mobilis* in 10 ml scale [8]. From this co-culture, 2.7 mg/ml of ethanol was produced from 10 mg/ml of cellulose. However, *C. thermocellum* was significantly inhibited at the low levels of ethanol, and leaving the undegraded cellulobiose in the co-culture inhibited the cell-associated cellobiase, which prevented the efficient conversion of cellulose to ethanol [8].

Recently, many researches have focused on the consolidated bioprocessing (CBP) for the simplification of the conversion process of cellulose to bioethanol [9,10]. The CBP was categorized into CBPs I and II. Category I CBP is an engineering method of a cellulase producer to be ethanologenic, while category II CBP of an ethanologen to be cellulolytic. A prototype model of CBP I is *Trichoderma reesei*[11] or *C. thermocellum*[12], which is one of the widely studied microorganisms because of producing several kinds of cellulases and β-glucosidases. These microorganisms can produce ethanol from cellulose, followed by the fermentation of the resulting sugars to ethanol in anaerobic growth conditions [13]. However, its ethanol yield, productivity, and ethanol tolerance are low due to the low expression of the relevant genes involved in ethanol fermentation or to the low activity of the enzymes encoded by these genes. These bottlenecks have to be solved to improve the feasibility of the CBP I microorganism.

The CBP II strain requires functional production and secretion of the variety of exoglucanases and endoglucanases, assimilation and fermentation on lignocellulose as a sole carbon source. Target microorganisms are engineered *E. coli* or *Z. mobilis* in bacteria, and *S. cerevisiae* in yeasts*.* Cellulolytic enzymes have been functionally expressed in some of yeasts [9], but the yeast has not been satisfied in anaerobic growth on cellulose. Recently, cellulases, xylanases, and amylases were expressed on the cell surface of *S. cerevisiae*[14-16] and 2.1 g/l of ethanol was produced from 10 g/l of phosphate-swollen amorphous cellulose using endoglucanase, cellobiohydrolase, and β-glucosidase displaying yeast [17]. Unfortunately, the expression of *T. reesei* cellobiohydrolases or exoglucanase, which play the critical role in cellulose degradation, is still poor in *S. cerevisiae*, and still remains lots of problems to be solved before a practical contribution to a worldwide energy supply.

In this study, one-pot bioethanol production system consisting of cellulase production from cellulose, saccharification of cellulose using cellulase in situ, and ethanol production was investigated. For one-pot ethanol production, understanding the concept of potential microorganisms to produce cellulase from cellulose and ethanol from hydrolysate is indispensable. Filamentous fungus *Acremonium cellulolyticus*, isolated in 1987 [18], was used in this study. The *A. cellulolyticus* produced cellulase from crystal cellulose [19], pretreated waste milk pack [20], and untreated waste paper sludge (PS) [21]. The cellulase activities of *A. cellulolyticus* were comparable to those of *T. reesei* origins, which was enough to proceed the bioconversion of waste office paper to L(+)-lactate [22] and gluconic acid [23]. For example, 160 g/l of cellulose contained in the waste PS was successfully converted to 40 g/l of ethanol in SSF using this cellulase and thermotolerant *S. cerevisiae*[24]. However, this kind of process is composed of two different and separate processes, such as cellulase and ethanol productions. A new emerging challenge of the one-pot ethanol process is to produce ethanol from cellulose in a single reactor using the co-culture of *A. cellulolyticus* and *S. cerevisiae*. *A. cellulolyticus* cells produce cellulase from cellulose, and the produced cellulase in situ saccharifies cellulose. *S. cerevisiae* cells consume the liberated reducing sugars and produce ethanol. Therefore, the combination of these different microorganisms has potentials for cellulase and ethanol productions.

This study is the first challenge for a practical application of ethanol production from cellulose in a single bioreactor using *A. cellulolyticus* and *S. cerevisiae* cells. *A. cellulolyticus* and *S. cerevisiae* cells grow in different media and different oxygen consumption for each other. For successful one-pot process for ethanol production, medium composition, times for inoculum of each microorganism and substrate addition should be carefully considered. This study demonstrated the high yield of ethanol production from biomass by optimizing these critical variables in one-pot bioethanol production.

## Results

*S. cerevisiae* inoculum time on co-culture of *A. cellulolyticus* and *S. cerevisiae* in a shake flask.

**Figure 1:**
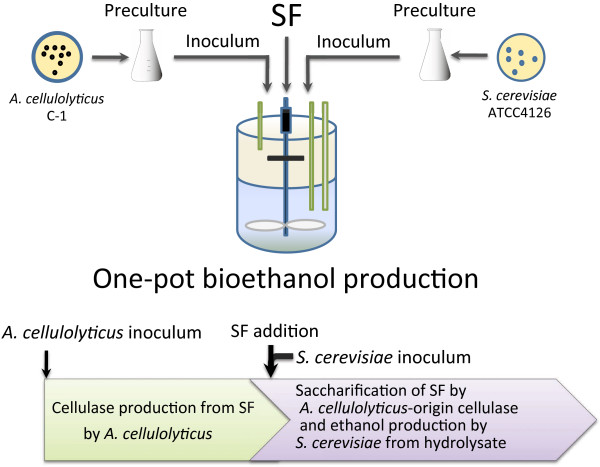
**Schematic diagram of one-pot bioethanol production.** SF was used as an inducing substrate for cellulase production by *A. cellulolyticus* C-1 at 28°C. (**A**) *A. cellulolyticus* cells produce cellulase from SF, and subsequently the produced cellulase saccharifies SF. *S. cerevisiae* cells consume the liberated glucose from the saccharification of SF and produce ethanol. The overall process from cellulase production to ethanol generation is carried out in a single reactor. (**B**) The first step of the one-pot bioethanol production is cellulase production by *A. cellulolyticus* cells and the second step is simultaneous saccharification of SF and ethanol production by the addition of *S. cerevisiae* inoculum and SF

**Figure 2:**
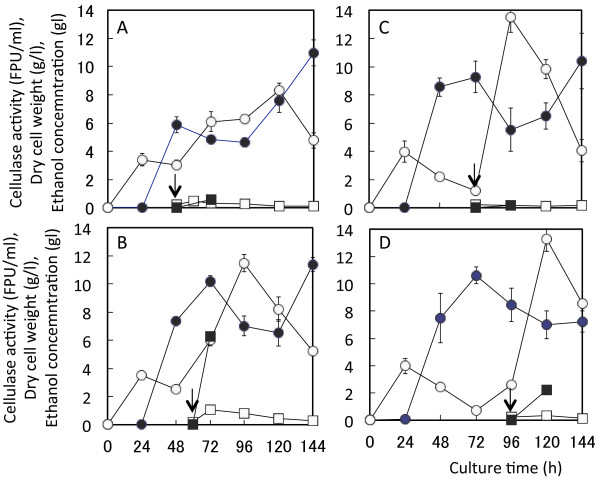
**Effect of the *S. cerevisiae *inoculum time and SF addition time on cellulase activity, DCW**_**A**_**, DCW**_**S**_**, and ethanol concentration in co-culture of *A. cellulolyticus *and *S. cerevisiae*.** Co-culture was carried out using cellulase producing-medium containing 2.5 g/l of yeast extract in a shake flask with shaking at 220 rpm. Ethanol production was started by additions of 10% inoculum of *S. cerevisiae* and 50 g/l of steam-sterilized SF as a powder at the culture time of 48 h (**A**), 60 h (**B**), 72 h (**C**), and 96 h (**D**). Symbols: open circles, DCW_A_; closed circles, cellulase activity; open squares, DCW_S_; closed squares, ethanol concentration. Arrows indicate the addition times. Error bars denote standard deviation (n = 3)

Using cellulase-producing medium, one-pot bioethanol production was carried out as shown in Figure 1. The 2.5 ml of *A. cellulolyticus* preculture and various inoculums of *S. cerevisiae* were co-cultured in cellulase producing-medium with SF as a cellulase-inducing substrate. DCW of *A. cellulolyticus* (DCW_A_) were in the range of 11.2–14.3 g/l (Additional file 1: Figure S1 A), and cellulase activity was 8–11.5 FPU/ml (Additional file 2: Figure S2 B). Cellulase activity and *A. cellulolyticus* cell growth did not show any co-operative inhibitory effects by the co-culture of *S. cerevisiae* and *A. cellulolyticus*, but *S. cerevisiae* cells did not grow at all (Additional file 1: Figure S1 C). This indicates that nutrients for *S. cerevisiae* growth were depleted because both *A. cellulolyticus* and *S. cerevisiae* cells consumed glucose liberated by saccharification of SF. In order to let *S. cerevisiae* cells grow after inoculation, residual glucose has to be present in culture. To decipher this optimal condition, co-culture was carried out with different *S. cerevisiae* inoculum time. When *S. cerevisiae* inoculum was added in the late exponential and stationary growth phases of *A. cellulolyticus* (Figure 2), cellulase activity was increased in the late exponential growth phase of *A. cellulolyticus* (48–60 h) and remained the highest activity in the stationary growth phase (72–96 h). Based on this finding, *S. cerevisiae* inoculum was added at four different culture times of 48, 60, 72, and 96 h. The cellulase activity was not affected by *S. cerevisiae* inoculum times of 48 and 60 h and maintained around 10 FPU/ml (Figure 2A and B). However, when the *S. cerevisiae* inoculum was added after the maximum cellulase activity, the cellulase activity decreased but DCW_A_ rebounded (Figure 2C and D). As *A. cellulolyticus* cells depleted nutrients, *S. cerevisiae* cells could not grow, resulting in below 1 g/l DCW of *S. cerevisiae* (DCW_s_) (Figure 2A–D). Ethanol concentration was 6.24 g/l at the inoculum time of 60 h (Figure 2B), and those at other inoculum times were below 2 g/l. Thus, to maximize the ethanol production in the co-culture of two different microorganisms, 60 h of inoculation time was determined.

### Medium preparation for one-pot bioethanol production in a shake flask

When both *A. cellulolyticus* and *S. cerevisiae* cells were co-cultured in a single reactor, DCW_S_ was at low levels (Additional file 1: Figure S1). Although cellulase production was followed by *S. cerevisiae* inoculum, DCW_S_ was still at low levels (Figure 2B). It means that it is difficult to get efficient production of ethanol in one-pot because of slow growth of *S. cerevisiae* due to depletion of nutrients. To promote the growth of *S. cerevisiae*, 0–5 g/l of yeast extract, 0–10 g/l of polypeptone, and their combined nutrients were added to cellulase-producing medium, respectively (Additional file 2: Figure S2). *A. cellulolyticus* cell growth was similar (Additional file 2: Figure S2 A) but cellulase activity was maintained higher than that without its addition (Additional file 2: Figure S2 B), suggesting that yeast extract and polypeptone were not inhibitory to the cellulase production in this co-culture. Ethanol production was increased to 40% by addition of 5 g/l of yeast extract compared to that without its addition (Additional file 2: Figure S2 C). Minimum requirement of yeast extract concentration for bioethanol production in *S. cerevisiae* was tested in the range of 0–7.5 g/l addition (Additional file 3: Figure S3). *A. cellulolyticus* cell growth and cellulase production were not affected by yeast extract addition (Additional file 3: Figure S3 A and B). The growth of *S. cerevisiae* was improved by 2–3 fold compared to that without its addition (Additional file 3: Figure S3 C). Since significant effect on ethanol production was not observed (Additional file 3: Figure S3 D), 2.5 g/l of yeast extract was added to cellulase-producing medium for stable growth of *S. cerevisiae* in the co-culture of two microorganisms. Thus, the minimal supplement of yeast extract significantly promoted the ethanol production as well as the growth of *S. cerevisiae*.

### Effect of temperature on cellulase production

**Figure 3:**
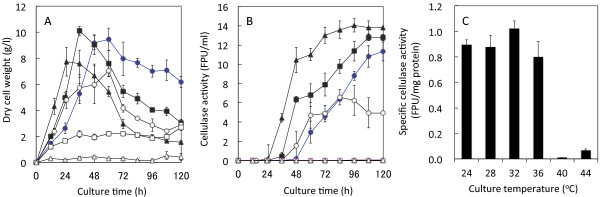
**Effect of the temperature in the culture of *A. cellulolyticus *C-1 on cell growth (A), cellulase activity (B), and specific cellulase activity (C).** Cellulase producing medium was used with SF as a sole inducing substrate for cellulase production at different culture temperatures (24, 28, 32, 36, 40, and 44°C). Symbols in **A** and **B**: closed circles, 24°C; closed squares, 28°C; closed triangles, 32°C; open circles, 36°C; open squares, 40°C; open triangles, 44°C. Error bars denote standard deviation (n = 3). As determined by ANOVA analysis, the cellulase activities affected by culture temperature in B are significant at *p* < 0.0001

In an ethanol production using the co-culture process, maintaining the cellulase activity in a high temperature without deactivation of the enzymatic activities is integral. Temperature is a critical factor for the stability of cellulase produced by *A. cellulolyticus*. Cellulase production was carried out in a wide range of culture temperatures. The maximum DCW_A_ at the cultures of 24 and 28°C were 9.4 and 10.1 g/l, respectively, but those at 32 and 36°C were below 8 g/l; at 40 and 44°C the *A. cellulolyticus* couldn’t grow (Figure 3A). Specific growth rates of *A. cellulolyticus* at 28 and 32°C were 0.07 and 0.49 h^-1^, respectively. Cellulase activity at the culture of 32°C reached to 14 FPU/ml, but that was 12.5 FPU/ml at 28°C, 11 FPU/ml at 24°C, 6 FPU/ml at 36°C, and 0 FPU/ml at 40 and 44°C (Figure 3B). Overall, the cellulase production rates at 28 and 32°C were 0.18 and 0.19 FPU/ml/h, respectively. There was no significant difference in cellulase activity between 28°C and 32°C, but specific cellulase activity was more than 1 FPU/mg protein at 32°C (Figure 3C). These results indicate that the optimal temperature for cellulase production is 32°C.

### Effect of agitation rate on the productions of cellulase and ethanol in co-culture

**Figure 4:**
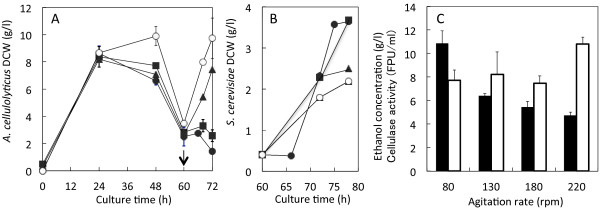
**Effect of the agitation rate on DCW**_**A **_**(A), DCW**_**S **_**(B), and ethanol concentration and cellulase activity (C) in co-culture of *A. cellulolyticus *and *S. cerevisiae*.** Ethanol production was carried out as the same method of Fig. 3, except for agitation rates, 80–220 rpm. Symbols in **A** and **B**: closed circles, 80 rpm; closed squares, 130 rpm; closed triangles, 180 rpm; open circles, 220 rpm. Closed and open bars in **C** denote ethanol concentration and cellulase activity, respectively. Arrow indicates inoculum and SF-addition time. Error bars denote standard deviation (n = 3). As determined by ANOVA analysis, the ethanol concentrations and cellulase activities affected by agitation rate in **D** are significant at *p* < 0.001 and *p* < 0.0001, respectively

Normally, the cellulase production by *A. cellulolyticus* cells was carried out in an aerobic condition whereas the ethanol production by *S. cerevisiae* cells in an anaerobic condition. To determine the logical conditions of oxygen supply, the effect of agitation rate on cellulase activity and ethanol production was investigated in a flask scale with different agitation rates (Figure 4). In the cellulase production phase the DCW_A_ reached 10 g/l at 220 rpm, but didn’t show significant change at 80 and 130 rpm (Figure 4A). On the other hand, the DCW_S_ in the ethanol production phase was tending to decrease with increased agitation rate (Figure 4B). The ethanol concentration was high at low agitation rate, but the cellulase activity was high at high agitation rate (Figure 4C). These findings suggest that the decreasing dissolved oxygen level followed by addition of *S. cerevisiae* inoculum is preferred for ethanol production in the co-culture of *A. cellulolyticus* and *S. cerevisiae*.

### One-pot bioethanol production from SF in a shake flask

**Figure 5:**
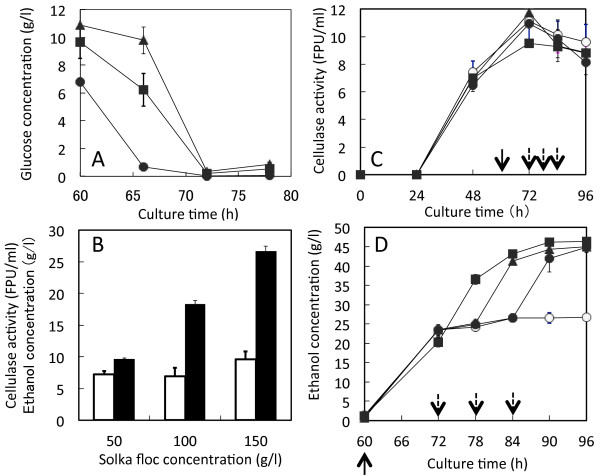
**One-pot bioethanol productions in a shake flask.** (**A**) and (**B**): Co-culture of *A. cellulolyticus* and *S. cerevisiae* was carried out with different SF concentration, 50, 100, and 150 g/l. (**C**) and (**D**): Co-culture of *A. cellulolyticus* and *S. cerevisiae* was carried out with 150 g SF/l concentration at the culture time of 60 h, and another 150 g SF/l was added at 72 h. Similar co-cultures were done with 150 g SF/l concentration at the culture time of 60 h, and another 150 g SF/l was added at 78 h or 84 h. Symbols in **A**: closed circles, 50 g/l SF; closed squares, 100 g/l SF; closed triangles, 150 g/l SF. Bars in **B**: open bars, cellulase activity; closed bars, ethanol concentration. Symbols in **C** and **D**: open circles, without addition; closed squares, addition time 72 h; closed triangles, addition time 78 h; closed circles, addition time 84 h. Arrows indicate SF-addition times. Error bars denote standard deviation (n = 3). As determined by ANOVA analysis, the ethanol productions by increased SF concentration in **B** and **D** are significant at *p* < 0.05 and *p* < 0.005

According to the determined conditions, one-pot bioethanol production was carried out with different initial SF concentrations from 50 to 150 g/l (Figure 5). DCW_A_ at 50, 100, and 150 g SF/l were 7.9, 8.0, and 8.3 g/l; DCW_S_ were 12.3, 13.0, and 12.5 g/l. The cell growth of *A. cellulolyticus* and *S. cerevisiae* did not show significant change. Residual glucose concentration at 50, 100, and 150 g SF/l were 6.8, 9.8, and 11 g/l, respectively (Figure 5A). During this process the cellulase activity remained at 7.5–8.5 FPU/ml without deactivation, but ethanol concentration was increased with increased SF concentration (Figure 5B). In addition, the *Y*_*e/SF*_s for 50, 100, and 150 g SF/l remained constant at 0.18 g/g, not affected by the initial amount of SF.

To improve the ethanol concentration, initial SF was 150 g/l at 60 h and another 150 g SF/l was added during ethanol fermentation at 72, 78, and 84 h (Figure 5C and D). The cellulase activity was 9–11.5 FPU/ml (Figure 5C), which was not affected by addition of SF during the ethanol production. Ethanol concentration was 26.7 g/l from 150 g SF/l, but it increased to 45–46.3 g/l by addition of 150 g/l SF (Figure 5D). *Y*_*e/SF*_s for addition 150 g SF/l at 72, 78, and 84 h were the same values of 0.15 g/g, respectively; overall ethanol production rate (*V*_e_), 0.45–0.48 g/l/h. This result indicates that cellulase activity remained enough to saccharify SF in the ethanol production phase and simultaneously *S. cerevisiae* cells were active in the ethanol fermentation phase.

### Improved one-pot bioethanol production from SF in fermentor

**Figure 6:**
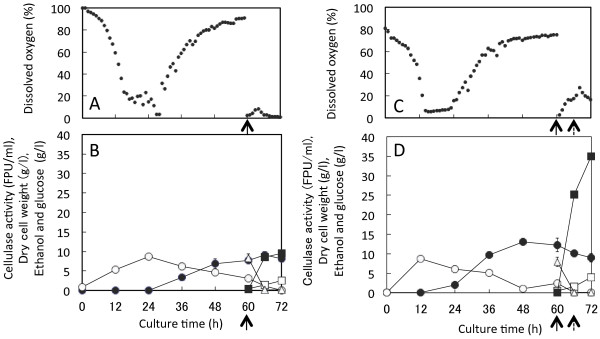
**One-pot bioethanol productions in a jar fermentor.** (**A**) and (**B**): Co-culture of *A. cellulolyticus* and *S. cerevisiae* was carried out with addition of 50 g SF/l at the culture time of 60 h. (**C**) and (**D**): Co-culture of *A. cellulolyticus* and *S. cerevisiae* was carried out with addition of 150 g SF/l at the culture times of 60 and 66 h. Agitation rate was 500 rpm during cellulase production, but dropped to 200 rpm at the culture time of 60 h for ethanol production. Symbols in **B** and **D**: open circles, DCW_A_; closed circles, cellulase activity; open triangles, glucose concentration; open squares, DCW_S_; closed squares, ethanol concentration. Arrows indicate SF-addition times. Error bars denote standard deviation (n = 3). As determined by ANOVA analysis, the ethanol productions in **B** and **D** are both significant at *p* < 0.0001

Jar fermentor was used to validate the one-pot bioethanol production using two microorganisms with 50 g SF/l. An agitation rate during cellulase production was kept at 500 rpm and at the culture time of 60 h, and decreased to 200 rpm during ethanol production phase, which resulted in the drop in dissolved oxygen level to 0% (Figure 6A). DCW_A_ was the highest at 24 h culture, and then cellulase activity increased to 9 FPU/ml (Figure 6B). When SF and *S. cerevisiae* inoculum were added, residual glucose concentration was 8.2 g/l, and dropped to 0 g/l after the culture of 66 h (Figure 6B). Ethanol concentration reached to 9.5 g/l at culture time of 72 h, and *Y*_*e/SF*_ was 0.19 g/g.

SF concentration was added another 150 g/l at the culture time of 66 h as the same cultural condition as Figure 6A and B. When the agitation rate was decreased to 200 rpm at the culture time of 60 h, the dissolved oxygen level was dropped to 0%, but increased gradually as high as 20% at the culture time of 66 h (Figure 6C). Cellulase activity was 12 FPU/ml at maximum and remained 10 FPU/ml during the ethanol production phase (Figure 6D). The ethanol concentration was increased sharply reaching 35.1 g/l (Figure 6D) at the culture time of 72 h, and the *Y*_*e/SF*_ and *V*_e_ values were 0.12 g/g and 0.49 g/l/h, respectively. Thus, one-pot bioethanol production from cellulose by two microorganisms is applicable to a jar fermentor scale production platform.

## Discussion

While the CBP is considered as the most ideal process, CBP is not useful in practice due to low ethanol concentration and its low yield. Alternatively, a simultaneous saccharification and fermentation (SSF) is an appropriate method for bioethanol production, but, in SSF, cellulase production process is excluded. Previous study reported that the ethanol concentration and yield based on initial cellulose were 36.5 g/l and 0.25 (g ethanol/g PS cellulose) from 150 g/l of cellulose contained in PS, respectively with cellulase loading of 15 FPU/g PS cellulose [24]. In the SSF, the total reaction time was 80 h, but when cellulase production process is included, it was at least 140 h, meaning that the *V*_e_ was 0.26 g/l/h.

In this one-pot bioethanol production process the productions of cellulase and ethanol were carried out by *A. cellulolyticus* and *S. cerevisiae*, respectively, in a single reactor. The total reaction time was dramatically reduced to 72 h including cellulase production, saccharification, and ethanol fermentation. During this process, the cellulase activity was not deactivated and remained constant (Figures 5B, 6B and D), enough to saccharify SF. The ethanol concentration was 25.6 g/l from 150 g SF/l with the *Y*_*e/SF*_ of 0.17 g/g, which was 70% comparing to that of the SSF. However, the *V*_e_ was 0.36 g/l/h, which is 1.4 fold to that of SFF. When 300 g/l SF was used in flask with cellulase loading of 25 FPU/g SF to increase ethanol concentration, the produced ethanol concentration, *Y*_*e/SF*_, and *V*_e_ were 46.3 g/l, 0.15 g/g, and 0.48 g/l/h, respectively (Figure 5D). However, in jar-fermentor, ethanol concentration, *Y*_*e/SF*_, and *V*_e_ were 35.1 g/l, 0.12 g/g, and 0.49 g/l/h, respectively (Figure 6D).

The *V*_e_ was improved 1.4 – 1.9-folds to that of SSF, indicating that the cellulase produced from *A. cellulolyticus* was highly stable and remained high activity without deactivation. The cellulase activity of firstly isolated *A. cellulolyticus* was only 5.0 FPU/ml using cellulose powder [18]. However, this activity was still insufficient for the saccharification of cellulolytic biomass industrially. We improved the cell line and optimized its medium for the practical production of cellulase and obtained 15.5 FPU/ml in the flask culture, 17.42 FPU/ml in the 7-L bioreactor, and 13.08 FPU/ml in the 50-L bioreactor [19]. *A. cellulolyticus* produces a complex mixture of cellulases, mainly comprised of four β-glucosidases (EC 3.2.1.21) and twelve distinct endo-cellulases/carboxymethyl cellulases (CMCases, EC 3.2.1.4) [18,25]. Other polysaccharide hydrolyzing enzymes, such as xylanases, amylases and β-1,3-glucanases are also present [26]. The most important enzyme in this mixture for the current process is an endo-cellulose type III-A that can produce glucose from cellulose without any participation of β-glucosidase [26]. In contrast, it is well known that the *Trichoderma* enzymes do not effectively saccharify cellulose alone because of their low β-glucosidase activity. When *A. cellulolyticus* cellulase and *Trichoderma* enzymes, GC220 were compared for saccharification of waste paper, their glucose contents among the hydrolysates were 83 and 72%, respectively [19]. Because the genus of *Trichoderma* generally produces relatively low β-glucosidase in its cellulolytic enzymes, those enzymes cannot convert cellulolytic biomass to glucose efficiently without addition of extraneous β-glucosidase. It is very important to have higher glucose contents in the hydrolysate since it is only glucose that *S. cerevisiae* can easily uptake.

Although this one-pot bioethanol production process significantly improve overall ethanol production rate, low *Y*_*e/SF*_ is still remained as an issue to be resolved. In the ethanol production phase *A. cellulolyticus* and *S. cerevisiae* cells consume glucose both for productions of cellulase and ethanol, respectively, and for their cellular maintenances, which cause the *Y*_*e/SF*_ decreased. It is better to keep in anaerobic condition in the ethanol production phase, but it was necessary to some extent agitation rate to avoid a precipitation of SF inside the reactor. To keep anaerobic condition, it may be effective to purge a nitrogen gas, but *A. cellulolyticus* cells cannot be alive. In this experiment, the dissolved oxygen level in the ethanol production phase increased to 20%, which might decrease the carbon flux from glucose to ethanol. It is necessary to optimize the dissolved oxygen both for maximizing ethanol production and for maintaining *A. cellulolyticus* cells actively. So far, this one-pot bioethanol production is an alternative strategy as a mimic of CBP, because cellulase production, saccharification of carbohydrate, and ethanol fermentation occur in a single reactor.

## Conclusion

This study establishes a method for practical one-pot bioethanol production from SF neither addition of extraneous cellulase nor pretreatment of cellulose. This one-pot bioethanol production includes cellulase production by *A. cellulolyticus*, saccharification of cellulose by cellulase in situ, and then ethanol production by *S. cerevisiae* from liberated reducing sugars in a single reactor. The potential of this process was also demonstrated in a stable and practical biorefinery using cellulosic biomass. Control of operational parameters, dissolved oxygen level, cellulose addition time, and *S. cerevisiae* inoculum time are still important for improving ethanol production in the co-culture with two different microorganisms. In particular, maintaining as higher than 20% and lower than 10% dissolved oxygen levels at cellulase and ethanol production phases, respectively, could be crucial for maximizing the one-pot bioethanol production. Further studies are planned to allow the improved ethanol yield from different cellulosic biomass and to be scaled up.

## Methods

### Raw materials

Solka-Floc (SF; CAS #9004-34-6; International Fiber Co., New York, USA) was used for cellulase and ethanol production. SF is a fine white powder comprised of approximately 70-80% crystalline cellulose and 20-30% amorphous cellulose. Medium components and other chemicals were purchased from Wako Pure Chem. Co. Ltd. (Tokyo, Japan) and stored at a room temperature.

### Microorganisms

*A. cellulolyticus* C-1 (Ferm P-18508), which is a hyper cellulase producer, and a mutant of wild type *A. cellulolyticus* Y-94, was provided by Tsukishima Kikai Co. Ltd. (Tokyo) [19]. *S. cerevisiae* ATCC 4126 (American Type Culture Collection, University Boulevard Manassas, VA, USA) was used for ethanol production in co-culture with *A. cellulolyticus* C-1.

### Culture media

The preculture medium for *A. cellulolyticus* is consisted (per liter) of 40 g SF, 24 g of KH_2_PO_4_, 1 ml of Tween 80 (MP Biomed. Co. Ltd., OH, USA), 5 g of (NH_4_)_2_SO_4_, 4.7 g of K_2_C_4_H_4_O_6_·4H_2_O, 1.2 g of MgSO_4_·7H_2_O, 10 mg of ZnSO_4_·7H_2_O, 9.28 mg of MnSO_4_·7H_2_O, 8.74 mg of CuSO_4_·7H_2_O and 2 g of urea (pH 4.0). The medium was sterilized at 121°C for 20 min, with separately sterilized ZnSO_4_·7H_2_O, MnSO_4_·7H_2_O and CuSO_4_·7H_2_O. Urea was sterilized by filtering through a 0.45 μm filter membrane (Toyo Roshi Kaisha Co. Ltd., Tokyo, Japan).

The preculture medium for *S. cerevisiae* is consisted (per liter) of 50 g glucose, 50 g/l YPD medium containing less than 0.04% of adenine (Sigma-Aldrich Co. Ltd., St. Louis, MO, USA). The YPD medium was composed of 20 g/l of bacteriological peptone, 10 g/l of yeast extract and 20 g/l of glucose.

### Co-culture of *A. cellulolyticus* and *S. cerevisiae*

Seed cultivation of *A. cellulolyticus* was carried out using 5 ml medium in a test tube at 28°C and 220 revolution per min (rpm) for 65 h. Cellulase production was carried out in 500 ml Erlenmeyer flask with 50 ml medium at 28°C and 220 rpm after addition of *A. cellulolyticus* inoculum with its volume fraction of 5%(v/v). The seed culture of *S. cerevisiae* was incubated for 24–30 h and by the time the cell density was about 2.2–2.8 g dry cell weight (DCW)/l. Co-culture was begun by addition of the desired inoculum sizes of *S. cerevisiae* and desired amount of SF into the culture of *A. cellulolyticus* at different addition times*.* The SF as a powder was used for a cellulase-inducing substrate and was steam-sterilized before use.

### Culture conditions of one-pot bioethanol production in a shake flask

To investigate medium components, culture temperature, agitation rate, and addition of *S. cerevisiae* inoculum for ethanol production in one-pot, co-culture was carried out in 500 ml Erlenmeyer flask with 50 ml medium. Medium is an important factor for producing both ethanol and cellulase with two different microorganisms. Preliminary test revealed yeast extract and polypeptone were the most effective nutrient at pH 4.5 and 28°C. Medium for *A. cellulolyticus* cultivation was added yeast extract, 0–7.5 g/l and used for productions of cellulase and ethanol. The effect of the culture temperature on cellulase production by *A. cellulolyticus* was tested in the temperature range of 24–44°C. To investigate the addition time of *S. cerevisiae* inoculum and SF for ethanol production, *S. cerevisiae* inoculum and SF were added at four different cellulase production phases, which was 48, 60, 72, and 96 h. *A. cellulolyticus* and *S. cerevisiae* requires different oxygen level, in aerobic and anaerobic growth pattern, respectively. Cellulase production in the culture of *A. cellulolyticus* was carried out in 500-ml Erlenmeyer flasks in a rotary shaker at an agitation rate of 220 rpm, but ethanol production in co-culture with *S. cerevisiae* with SF was carried out at different agitation rates of 80–220 rpm. After the addition of *S. cerevisiae* inoculum and SF, ethanol concentration and cellulase activity were measured.

### One-pot bioethanol production in a jar fermentor

Three liters jar fermentor (MDL-80, Marubishi, Tokyo Japan) with a 1.67 l working volume was used. One hundred and fifty millilitres of seed culture of *A. cellulolyticus* were added to 1350 ml of cellulase production medium supplemented with nutrients, and then cultured at agitation rate of 500 rpm and aeration rate of 1.5 volume per volume per min (vvm). Co-culture was begun by adding a 10%(v/v) inoculum of *S. cerevisiae* and desired amount of SF as a powder into the culture of *A. cellulolyticus* at the desired culture time*.* After the addition of *S. cerevisiae* inoculum and SF, agitation rates decreased to 200 revolution per min (rpm). After culture, DCW of *A. cellulolyticus* (DCW_A_) and *S. cerevisiae* (DCW_S_) was measured. The culture broth was centrifuged at 9447 g, and the supernatant was stored in a 4°C refrigerator before the measurement of a cellulase activity and ethanol and protein concentrations.

For the practical ethanol production from SF, the ethanol yield based on the initial SF (*Y*_*e/SF*_, g ethanol/g SF) and an overall ethanol production rate (*V*_e_, g/l/h) are defined as follow:

(1) $$Y_{e/SF}=\Delta C_{e}/C_{\mathrm{SF}}$$

(2) $$V_{\text{e}}=\Delta C_{e}/t_{\text{one}-\text{pot}}$$

where, Δ*C*_*e*_ and *C*_*SF*_ denote concentrations of produced ethanol and initial SF, respectively, and *t*_one-pot_ indicates culture time including cellulase production, saccharification, and fermentation.

### Analytical methods

Due to the difficulty in separating the mycelia of *A. cellulolyticus* C-1 from *S. cerevisiae* cells during the co-culture, total intracellular nucleic acid concentration (*INA*) was measured [27] and converted to total DCW (DCW_t_) as follows [19].

(3) $$INA\left(\text{g}/\text{l}\right)=1.72\;\times \;\text{absorbance at}\;260\;\text{nm}$$

(4) $${\text{DCW}}_{\text{t}}\left(\text{g}/\text{l}\right)=16.565\times INA\text{.}$$

However, since the INA value contained *S. cerevisiae* and *A. cellulolyticus* cell masses, DCW of *A. cellulolyticus* (DCW_A_) was calculated as follows.

(5) $${\text{DCW}}_{\text{A}}={\text{DCW}}_{\text{t}}–{\text{DCW}}_{\text{S}}$$

where, DCW_S_ denotes DCW of *S. cerevisiae*.

During the co-culture, the number of *S. cerevisiae* was counted and converted to DCW_S_. A culture broth of the co-culture was diluted, stained with 0.4% trypan blue and its number was counted with hemacytometer (Hirschmann Em Techcolor, Eberstadt, Germany). The DCW_S_ was determined using a calibration curve between DCW_S_ and *S. cerevisiae* cell number. The harvested cells in the culture without solid components were re-suspended in distilled water and centrifuged again to remove medium components. The precipitate was dried at 105°C. Correlation of DCW_S_ and the cell number was as follow,

(6) $${\text{DCW}}_{\text{S}}\left(\text{g}/\text{l}\right)=1\times 10^{-8}\times \text{cell number}\left(\text{cells}/\text{ml}\right)+0.3431$$

Cellulase activity was measured using the standard IUPAC procedure with Whatman No. 1 filter paper, and the activity was expressed in filter paper unit (FPU). The FPU unit is based on the International Unit (IU) in which the absolute amount of glucose at a critical dilution is 2 mg for 0.5 ml critical enzyme concentration for 60 min [28].

Reducing sugar was measured by dinitrosalicylic acid (DNS) [29]. Residual glucose concentration was measured by a biochemistry analyzer (2700 SELECT, YSI Life Sci., Yellow Springs, OH, USA).

Ethanol concentration was measured using a Gas Chromatography (Shimadzu-2014, Shimadzu Co. Ltd., Tokyo, Japan) using a packed column (Gaskuropack5460/80, GC−2014Glass ID.3.2ϕ × 2.1 m, GL Science Co. Ltd., Tokyo, Japan), with the following operational conditions: temperature of column and detector were 110 and 250°C, respectively, nitrogen gas flow rate 60 ml/min and the injected sample volume 2 μl.

The total soluble protein concentration in crude and enzyme solutions was measured by the Lowry method [30]. Protein standard was prepared using bovine serum albumin.

### Statistical analysis

Statistical analysis of experimental data was performed by a factorial ANOVA, using the least significant difference by the STATISTICA software package (Ver 5.5, Tulsa, OK, USA).

## Abbreviations

ANOVA: analysis of variance; CBP: consolidated bioprocess; DCW: dry cell weight; DCW_A_: dry cell weight of *A. cellulolyticus*; DCW_S_: dry cell weight of *S. cerevisiae*; DCW_t_: dry cell weight of *A. cellulolyticus* and *S. cerevisiae*; PS: paper sludge; SF: Solka-Floc; SSF: simultaneous saccharification and fermentation: *V*_e_, overall ethanol production rate in one-pot bioethanol production process (g/l/h): *Y*_*e/SF*_, the ethanol yield based on the initial SF (g ethanol/g SF).

## Competing interests

The authors declare that they have no competing interests.

## Authors’ contributions

EYP directly supervised the project, participated in its experimental design, data interpretation, and was responsible for writing the manuscript. KN carried out the co-culture, the hydrolysis and fermentation experiments. TK participated in handling and discussion of co-culture of *A. cellulolyticus* and *S. cerevisiae* cells. All authors have read and approved the manuscript.

## Supplementary Material

Additional file 1 **Figure S1. DCW of *A. cellulolyticus *(A), cellulase production (B), and *S. cerevisiae *cell number (C) in co-culture of *A. cellulolyticus *and *S. cerevisiae *.** Various *S. cerevisiae* inoculums were added to 2.5 ml of *A. cellulolyticus* preculture in 500 ml Erlenmeyer flask with working volume of 50 ml, and were co-cultured at 28°C for 120 h. Inoculum sizes of *S. cerevisiae* in A and B were 2.5% (closed circles), 5.0% (open squares), 5.75% (open triangles), and 10.0% (open circles). *S. cerevisiae* cell number in C was measured at 24 h (open bars) and 48 h (closed bars). Error bars denote standard deviation (n=3). As determined by ANOVA analysis, the cellulase activities affected by *S. cerevisiae* inoculum sizes in B are *p* < 0.001. Click here for file

Additional file 2 **Figure S2. Effect of nutrients addition on DCW**_**A **_**(A), cellulase activity (B), and ethanol production (C) in co-culture of *A. cellulolyticus *and *S. cerevisiae*.** Ethanol production was carried out with addition of 50 g SF/l and 10% inoculum at the culture time of 60 h with agitation rate of 220 rpm. Used medium was cellulase producing-medium containing various ratios of yeast extract and polypeptone, without its addition (closed squared); 5 and 10 g/l (closed triangles); 2.5 and 5 g/l (open circles); 5 and 0 g/l (open squares); 0 and 10 g/l (open triangles). Arrows indicate inoculum and SF-addition time. Error bars denote standard deviation (n=3). As determined by ANOVA analysis, the cellulase activities (B) and ethanol concentrations (C) affected by yeast extract are *p* value of 0.0004 and 0.0027, respectively. Click here for file

Additional file 3 **Figure S3. Effect of yeast extract on DCW**_**A **_**(A), cellulase activity (B), and DCW**_**S **_**(C), ethanol production (D) in co-culture of *A. cellulolyticus *and *S. cerevisiae*.** Ethanol production was carried out with addition of 50 g SF/l and 10% inoculum at the culture time of 60 h with agitation rate of 220 rpm. Used medium was cellulase producing-medium containing various concentrations of yeast extract, without addition (closed circles); 2.5 g/l (closed squares); 5 g/l, (closed triangles); 7.5 g/l (open circles). Arrows indicate inoculum and SF-addition time. Error bars denote standard deviation (n=3). As determined by ANOVA analysis, the cellulase activities and ethanol concentrations affected by yeast extract concentration in B and D are both significant at *p* < 0.0001, respectively. Click here for file
