# Supplementary figures and images for: One-pot bioethanol production from cellulose by co-culture of Acremonium cellulolyticus and Saccharomyces cerevisiae
Source: Biotechnol Biofuels. 2012 Aug 31;5:64. doi: 10.1186/1754-6834-5-64 (PMC3493283; doi:10.1186/1754-6834-5-64)

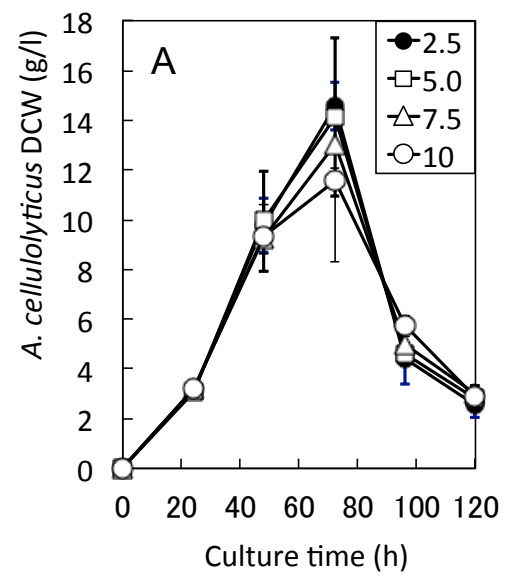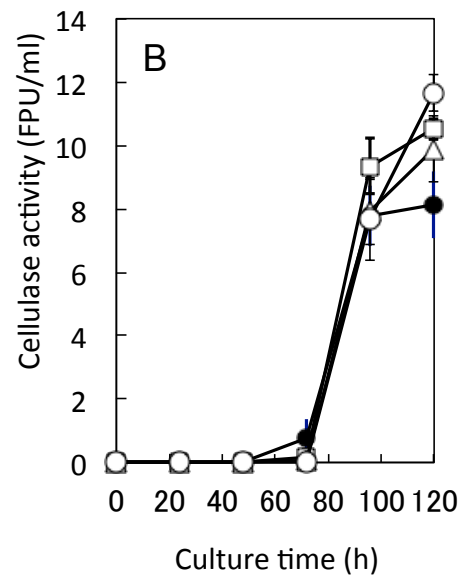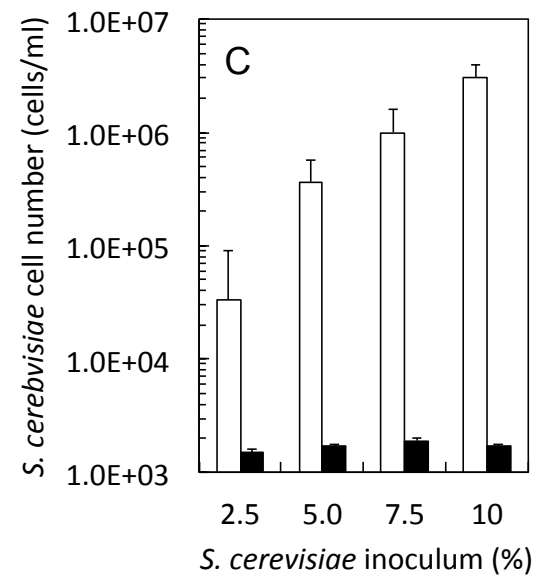

Supplement: Additional file 1 — Figure S1. DCW of A. cellulolyticus (A), cellulase production (B), and S. cerevisiae cell number (C) in co-culture of A. cellulolyticus and S. cerevisiae . Various S. cerevisiae inoculums were added to 2.5 ml of A. cellulolyticus preculture in 500 ml Erlenmeyer flask with working volume of 50 ml, and were co-cultured at 28°C for 120 h. Inoculum sizes of S. cerevisiae in A and B were 2.5% (closed circles), 5.0% (open squares), 5.75% (open triangles), and 10.0% (open circles). S. cerevisiae cell number in C was measured at 24 h (open bars) and 48 h (closed bars). Error bars denote standard deviation (n=3). As determined by ANOVA analysis, the cellulase activities affected by S. cerevisiae inoculum sizes in B are p < 0.001. [file 1754-6834-5-64-S1.pdf]

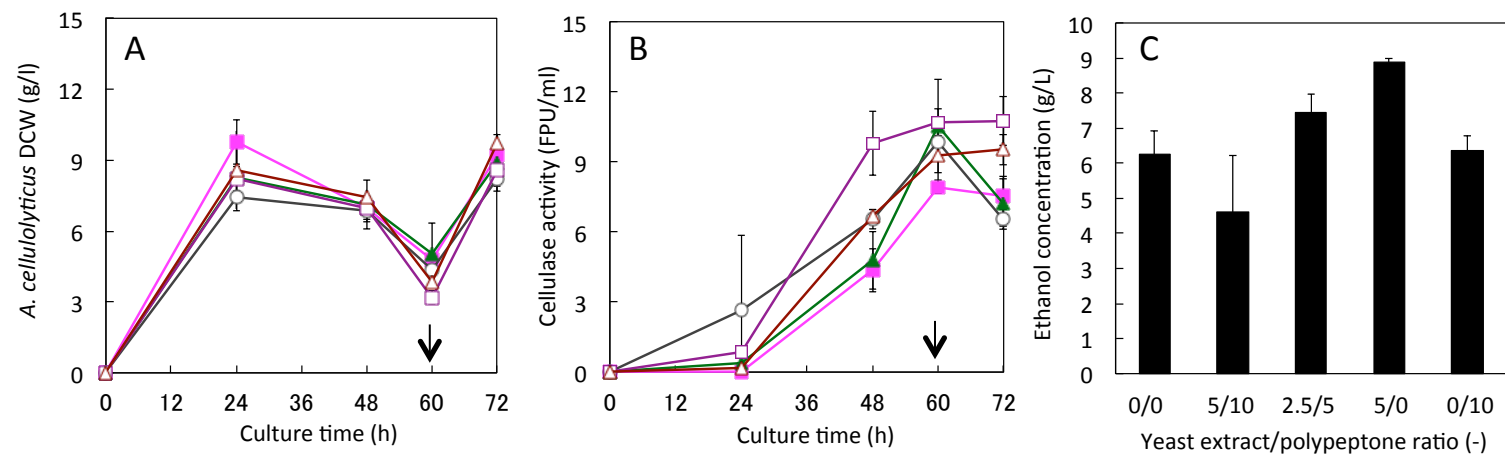

Supplement: Additional file 2 — Figure S2. Effect of nutrients addition on DCWA (A), cellulase activity (B), and ethanol production (C) in co-culture of A. cellulolyticus and S. cerevisiae. Ethanol production was carried out with addition of 50 g SF/l and 10% inoculum at the culture time of 60 h with agitation rate of 220 rpm. Used medium was cellulase producing-medium containing various ratios of yeast extract and polypeptone, without its addition (closed squared); 5 and 10 g/l (closed triangles); 2.5 and 5 g/l (open circles); 5 and 0 g/l (open squares); 0 and 10 g/l (open triangles). Arrows indicate inoculum and SF-addition time. Error bars denote standard deviation (n=3). As determined by ANOVA analysis, the cellulase activities (B) and ethanol concentrations (C) affected by yeast extract are p value of 0.0004 and 0.0027, respectively. [file 1754-6834-5-64-S2.pdf]

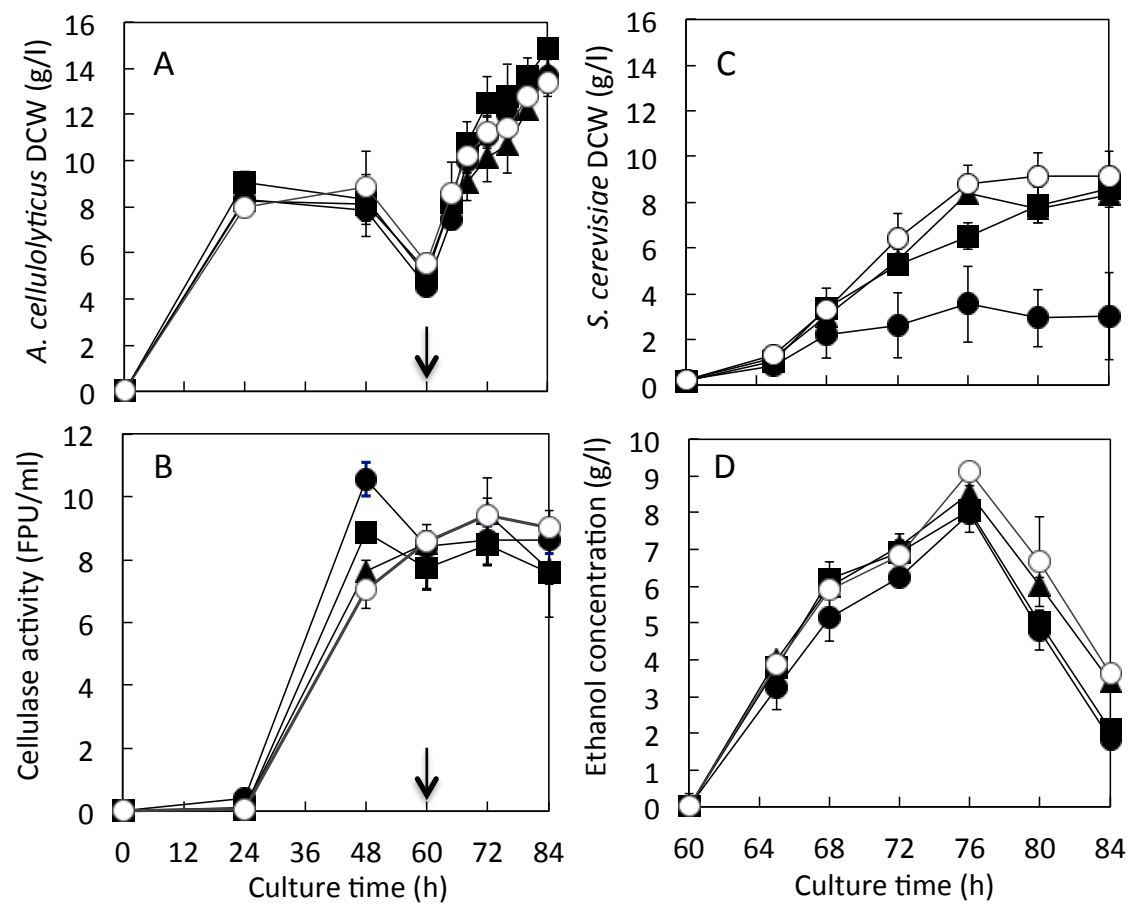

Supplement: Additional file 3 — Figure S3. Effect of yeast extract on DCWA (A), cellulase activity (B), and DCWS (C), ethanol production (D) in co-culture of A. cellulolyticus and S. cerevisiae. Ethanol production was carried out with addition of 50 g SF/l and 10% inoculum at the culture time of 60 h with agitation rate of 220 rpm. Used medium was cellulase producing-medium containing various concentrations of yeast extract, without addition (closed circles); 2.5 g/l (closed squares); 5 g/l, (closed triangles); 7.5 g/l (open circles). Arrows indicate inoculum and SF-addition time. Error bars denote standard deviation (n=3). As determined by ANOVA analysis, the cellulase activities and ethanol concentrations affected by yeast extract concentration in B and D are both significant at p < 0.0001, respectively. [file 1754-6834-5-64-S3.pdf]
